# Calcium-dependent cyto- and genotoxicity of nickel metal and nickel oxide nanoparticles in human lung cells

**DOI:** 10.1186/s12989-018-0268-y

**Published:** 2018-07-17

**Authors:** Sebastiano Di Bucchianico, Anda R. Gliga, Emma Åkerlund, Sara Skoglund, Inger Odnevall Wallinder, Bengt Fadeel, Hanna L. Karlsson

**Affiliations:** 1 Institute of Environmental Medicine, Karolinska Institutet, Stockholm, Sweden; 2 KTH Royal Institute of Technology, Department of Chemistry, Surface and Corrosion Science, Stockholm, Sweden

**Keywords:** Nickel/nickel oxide nanoparticles, Chromosomal aberrations, Endoreduplication, Calcium homeostasis, Carcinogenic potential

## Abstract

**Background:**

Genotoxicity is an important toxicological endpoint due to the link to diseases such as cancer. Therefore, an increased understanding regarding genotoxicity and underlying mechanisms is needed for assessing the risk with exposure to nanoparticles (NPs). The aim of this study was to perform an in-depth investigation regarding the genotoxicity of well-characterized Ni and NiO NPs in human bronchial epithelial BEAS-2B cells and to discern possible mechanisms. Comparisons were made with NiCl_2_ in order to elucidate effects of ionic Ni.

**Methods:**

BEAS-2B cells were exposed to Ni and NiO NPs, as well as NiCl_2_, and uptake and cellular dose were investigated by transmission electron microscopy (TEM) and inductively coupled plasma mass spectrometry (ICP-MS). The NPs were characterized in terms of surface composition (X-ray photoelectron spectroscopy), agglomeration (photon cross correlation spectroscopy) and nickel release in cell medium (ICP-MS). Cell death (necrosis/apoptosis) was investigated by Annexin V-FITC/PI staining and genotoxicity by cytokinesis-block micronucleus (cytome) assay (OECD 487), chromosomal aberration (OECD 473) and comet assay. The involvement of intracellular reactive oxygen species (ROS) and calcium was explored using the fluorescent probes, DCFH-DA and Fluo-4.

**Results:**

NPs were efficiently taken up by the BEAS-2B cells. In contrast, no or minor uptake was observed for ionic Ni from NiCl_2_. Despite differences in uptake, all exposures (NiO, Ni NPs and NiCl_2_) caused chromosomal damage. Furthermore, NiO NPs were most potent in causing DNA strand breaks and generating intracellular ROS. An increase in intracellular calcium was observed and modulation of intracellular calcium by using inhibitors and chelators clearly prevented the chromosomal damage. Chelation of iron also protected against induced damage, particularly for NiO and NiCl_2_.

**Conclusions:**

This study has revealed chromosomal damage by Ni and NiO NPs as well as Ni ionic species and provides novel evidence for a calcium-dependent mechanism of cyto- and genotoxicity.

**Electronic supplementary material:**

The online version of this article (10.1186/s12989-018-0268-y) contains supplementary material, which is available to authorized users.

## Background

Exposure to particles containing nickel (Ni) via inhalation is common at occupational settings such as in nickel refineries, stainless steel production sites and at work places where welding is performed. Furthermore, considerable evidence shows that such exposure increases the risks of both lung fibrosis and cancer in exposed workers [1, 2]. The International Agency for Research on Cancer has therefore classified nickel compounds as carcinogenic to humans (Group 1) whereas Ni metal, on the other hand, is classified as Group 2B (possibly carcinogenic to humans) [3, 4]. This is due to a lack of associations observed in epidemiological studies and no clear association between respiratory tumors and micron-sized nickel metal powder in a chronic inhalation study on rats [5]. Recently, IARC also concluded that there now is sufficient evidence in humans that welding fumes cause lung cancer [6]. Nickel compounds are categorized as water-soluble or water-insoluble (poorly soluble), or alternatively grouped as soluble, sulfidic and oxidic Ni [7]. Indeed, the toxicological profile appears to differ substantially between these groups. When, for example, soluble nickel sulfate (NiSO_4_), green nickel oxide (NiO) and nickel subsulfide (Ni_3_S_2_) were tested in two-year animal inhalation studies, an increase of lung tumors in rats was found for NiO and Ni_3_S_2_ (most potent), but not for NiSO_4_ [8]. One plausible explanation is that soluble Ni is relatively quickly flushed from the lung tissue and, in addition, the cellular uptake appears to be rather limited, which results in less carcinogenic effects in vivo and in human epidemiologic studies [9]. In contrast, poorly soluble Ni compounds are able to enter cells by phagocytosis and/or macropinocytosis and the efficiency of the uptake depends on factors such as size, crystalline structure and surface characteristics (charge, shape, etc.) [9]. Once inside cells and in acidified cytoplasmic vacuoles, such Ni-containing particles can dissolve and release nickel ions, and it has been suggested that this intracellular dissolution allows Ni ions/species to enter the nucleus [10]. This has resulted in a Ni-bioavailability model, which proposes that the bioavailability of released nickel species in the nucleus of epithelial respiratory cells may explain current findings on the carcinogenic potential of nickel-containing particles [11]. This, in turn, depends also on the clearance governing the maximum retained dose. The model was elaborated based on data for micron-sized Ni-containing particles, and its applicability to estimate the carcinogenic potential of Ni-containing nanoparticles (NPs) still remains to be explored. Ni and NiO NPs are manufactured to be used e.g. as catalysts, sensors, antimicrobials and in energy storage devices [12]. The number of humans exposed to manufactured Ni and NiO NPs is likely still limited, but two case reports have indicated severe effects following inhalation [13, 14]. Pronounced inflammatory effects have been observed following exposure to NiO NPs in animal studies [15, 16], and several in vivo and in vitro studies have shown NPs to induce more toxicity as compared with micron-sized Ni-containing particles [17–20]. Particle size is, however, not the only parameter governing the detrimental response and nickel release as well as toxicity has also been observed for reactive micron-sized Ni particles [21, 22].

Despite the classification of Ni compounds as carcinogenic, and Ni metal as possible carcinogenic, the molecular mechanisms leading to lung cancer are not fully elucidated. A wide range of possible mechanisms has been proposed including genotoxicity, effects on DNA repair proteins and epigenetic changes [2, 23]. Even though some studies have investigated the ability of Ni and NiO NPs to induce DNA strand breaks [22, 24], more in-depth genotoxicity studies are still needed and mechanisms to be elucidated. To this end, the aim of this study was to investigate the ability of Ni and NiO NPs to alter genome stability in human bronchial epithelial BEAS-2B cells and to explore the underlying mechanisms. In particular, we focused on formation of micronucleus and chromosomal aberration, hallmarks of genome instability and predictors of cancer risk, and elucidated the role of oxidative stress as well as calcium and iron homeostasis. The cell uptake/cell dose was analyzed using inductively coupled plasma mass spectrometry (ICP-MS) and comparisons were made with soluble nickel (NiCl_2_) in order to elucidate effects of nanoparticles vs ionic species. We used BEAS-2B cells for several reasons including; 1) they have been suggested to exhibit high similarities in gene expression pattern with primary cells and are therefore regarded a good lung model [25]; 2) they are cultured under serum free conditions that is more relevant for lung cells and could constitute a more sensitive model for NPs [26]; 3) they have been widely used for investigating genotoxic effects of NPs (see e.g. [27]).

## Methods

### Nanoparticles

Nickel nanopowder (Ni, < 100 nm, purity > 99%, Cat#: 577995, 93397KJ), nickel (II) oxide nanopowder (NiO, < 50 nm, > 99.8% purity, Cat# 637130, 17198PJ) and water soluble nickel (II) chloride (NiCl_2_·6H_2_O, Cat# N5756, 37H3494) were purchased from Sigma-Aldrich. Size and morphology of the same batch of Ni and NiO NPs have previously been reported [22] using transmission electron microscopy (TEM) showing Ni NPs to be predominantly less than 100 nm with a BET-area of 6.41 m^2^/g and the NiO NPs less than 50 nm and a BET-area of 102 m^2^/g. Particle dispersions were freshly prepared in cell medium (1 mg/mL) followed by 15 min sonication in an ultrasonic bath (Branson 2200). Subsequent dilutions were immediately prepared in cell medium prior to exposure. Previous modelling of speciation of released ions in cell medium showed that Ni ions form strong complexes with amino acids and are not present as free ions or labile complexes [22].

### Surface analysis using XPS

X-ray photoelectron spectroscopy (XPS, UltraDLD spectrometer, Kratos Analytical, Manchester, UK) was utilized in order to characterize the outermost surface of Ni and NiO nano. High resolution spectra (20 eV) were acquired using a monochromatic Al Kα-source (1486.6 eV, 300 W). Charge corrections were made to C 1 s at 285.0 eV.

### Cell culture and exposures

The immortalized human bronchial epithelial cell line (BEAS-2B, European Collection of Cell Cultures) was cultured in supplemented bronchial epithelial cell growth medium (BEGM, Lonza) on pre-coated flasks according to previously described [28]. It is noted that BEGM is serum-free and specially designed to support the growth of bronchial epithelial cells. BEAS-2B cells were independently authenticated according to ISO 9001–2004 and Mycoplasma tested (Leibniz Institute DSMZ, Germany). BEAS-2B cells were seeded in 6- or 24-well plates at an approximate density of 5 × 10^4^ cells/cm^2^. After 24 h, particle dispersions were directly added to the cell cultures to obtain a final mass concentration of 1, 5 and 10 μg/mL based on mass of nickel, corresponding to 0.21, 1.05 and 2.1 μg Ni/cm^2^. The final volume used in 6- or 24-well plates was 2 mL and 0.4 mL, respectively, in order to maintain the same μg/cm^2^ between exposures. The doses were selected mainly based on cytotoxicity with the intention to use doses that were non-cytotoxic or showing effects of maximum around 30% cytotoxicity. According to the OECD guideline for in vitro MN assay (487), care should be taken not to exceed 60% cytotoxicity because higher levels may induce micronuclei as a secondary effect of cytotoxicity.

### PCCS analysis

Dynamic light scattering using photon cross-correlation spectroscopy (PCCS) (NanoPhox, Sympatec, Germany) was used to determine the hydrodynamic size of the NPs (10 μg/mL) in cell medium. In brief, 1 mL dispersions of Ni and NiO (10 μg/mL) were prepared in cuvettes and analyzed directly after dispersion (0 h), after 2 and 24 h. Triplicate samples were analyzed to identify the size distribution pattern and data is presented based on single sample analysis measured three times each. Calibration was made using standard latex samples (20 ± 2 nm) and blank samples were analyzed prior to all measurements [28].

### TEM analysis

TEM imaging was performed as previously described [28]. In brief, BEAS-2B cells were exposed to 10 μg Ni/mL for 48 h. They were then washed, harvested, centrifuged and fixed in freshly prepared 0.1 M glutaraldehyde solution. The pellets were then post fixed in 2% osmium tetroxide. Ultrathin sections (approximately 60–80 nm) were cut and contrasted with uranyl acetate followed by lead citrate. Samples were then examined using a FEI Tecnai 12 Spirit Bio TWIN transmission electron microscope at 100 kV.

### ICP-MS analysis

The cellular uptake and Ni release from the particles in cell medium was quantified using inductively coupled plasma mass spectrometry (ICP-MS). For cellular uptake studies, BEAS-2B cells were seeded in 6-well plates as previously described and exposed to Ni, NiO and NiCl_2_ at the following concentrations and time points: 10 μg Ni/mL for 4 h; 10 μg Ni/mL for 24 h; 1, 5, 10 μg Ni/mL for 48 h. At the end of the exposure the cells were washed two times with PBS, harvested and counted. For Ni release in cell medium, Ni and NiO NPs were incubated with BEGM cell medium for 48 h, at 37 °C. After incubation, the particle dispersions were centrifuged at 13,000 rpm, 1 h (0 °C) and the supernatants were carefully collected. Non-centrifuged dispersions were also collected in order to measure the amount of Ni added. All samples (ion release, cellular uptake) were acidified in 32% HNO_3_ for at least 48 h. Thereafter the samples were diluted to reach approximately 2% HNO_3_. For all samples ^58^Ni and ^60^Ni isotopes were quantified using an iCAP Q ICP-MS (Thermo Scientific) instrument in KED mode. Calibration standards of 1, 5, 10, 50, 100, 500 μg/L Ni were prepared using a 1000 ppm reference standard (Spectrascan). Samples spiked with 5 μg/L indium were used as an internal standard with a range of recovery between 80 and 105%. Each sample was injected at least 3 times and the RSD acceptance was set at 15%. Cell uptake results were normalized according to the cell number and expressed as pg Ni/cell considering the average values of the analyzed isotopes. Nickel release was expressed as percentage Ni released in cell medium in relation to the amount of added Ni, based on the average values of the analyzed isotopes. The limits of detection for the investigated isotopes were calculated from the SD of the untreated control (3 x SD of control) and estimated as < 0.013 μg/L (^58^Ni), < 0.008 μg/L (^60^Ni) for the cellular uptake experiments and < 0.012 μg/L (^58^Ni), < 0.020 μg/L (^60^Ni) for the nickel release experiments.

### Annexin V-FITC/PI analysis

The annexin V-FITC/propidium iodide (PI) kit (Calbiochem) was used following the manufacturer’s instructions for detection of apoptosis and necrosis [29]. BEAS-2B cells were exposed in 6-well plates to 1, 5 and 10 μg Ni/mL of Ni, NiO NPs and NiCl_2_ for 48 h. Live, apoptotic and necrotic cells were detected with a BD Accuri™ C6 Cytometer (BD Biosciences) and twenty thousand events were collected and analyzed with the BD Accuri™ software template. Cell debris was gated out before analysis based on light-scattering properties. Untreated cells and 0.2 μg/mL etoposide (Sigma-Aldrich) were used as negative and positive controls.

### Comet assay

DNA damage (single and double DNA strand breaks, and alkali labile sites) was evaluated by the alkaline version (pH > 13) of comet assay. BEAS-2B cells were seeded in 24-well plates and exposed for 48 h after which the comet assay was performed as described in [28]. Untreated cells were used as negative control and cells treated with 25 μM H_2_O_2_ (Sigma-Aldrich) for 10 min on ice as positive control.

### Intrinsic and intracellular ROS

The 2′,7′-dichlorodihydrofluorescein diacetate (DCFH-DA) assay was used to measure intrinsic and intracellular ROS production similar as previously described [24]. In short, the intrinsic ROS production was measured by first cleaving the diacetate using NaOH. After neutralization, the Ni was added in final concentrations of 10, 25 and 50 μg Ni/mL in a black 96 well plate with or without HRP (horseradish peroxidase, final concentration 2.2 U/mL). Fluorescence was then measured (excitation 485 nm, emission 535 nm) using a plate reader (Tecan Infinite F200). For the intracellular ROS, BEAS-2B cells were seeded in black 96-well plates with transparent bottom and were after 24 h loaded with 20 μM DCFH-DA in HBSS (Hank’s buffered salt solution) for 30 min at 37 °C. Subsequently, cells were washed with HBSS and exposed to 1, 5 and 10 μg Ni/mL of Ni, NiO NPs and NiCl_2_. Tert-butyl hydroperoxide (TBP, 10 and 30 μM) was used as positive control. Fluorescence was recorded every 5 min over 2 h (excitation 485 nm, emission 535 nm) using a plate reader (Tecan Infinite F200) at 37 °C and ROS increase was calculated as mean slope per min and normalized to the unexposed control. Results are presented as mean values ±standard error of mean (SEM) of three independent experiments.

### Intracellular calcium

BEAS-2B cells were seeded in 6-well plates and exposed to 5 μg Ni/mL of Ni, NiO NPs and NiCl_2_ for 2 h. The cells were then washed twice in Ca/Mg free HBSS (Gibco) and incubated with 5 μM Fluo-4-acetoxymethyl ester (Fluo-4 AM, ThermoFicher Scientific, in 3% DMSO) in Ca/Mg free HBSS at 37 °C for 30 min. The cells were then washed twice in HBSS and detached from the plates using Ca/Mg free cell dissociation buffer (Gibco) for 30 min at 37 °C. The cells were then immediately analyzed with the flow cytometer BD Accuri C6 (BD Biosciences) using the 530/30 filter. The fluorescence in 10,000 cells/sample was recorded and addition of 2.5 μM calcium ionophore A23187 (in DMSO, Sigma-Aldrich) was used as a positive control. Data analysis was performed using the BD Accuri C6 software and the median fluorescence of each sample was recorded. Results are presented as mean values ± SEM of three independent experiments. Student’s t-test was used to analyze difference between control and exposed cells.

### Micronucleus assay

Cytotoxicity, cytostasis and chromosomal damage were studied by cytokinesis-block micronucleus cytome assay (CBMN Cyt) (OECD 487**)**. BEAS-2B cells were exposed and after 20 h, 5 μg/mL cytochalasin-B (Sigma-Aldrich) was used to block cytokinesis in a delayed co-treatment. Cells were harvested after additional 28 h culture (48 h exposure), treated 1 min with 0.075 M KCl (hypotonic solution), washed twice with fresh cold fixative (methanol:acetic acid 6:1) and placed at − 20 °C overnight. The cell solutions were placed onto clean and cold glass slides and the air-dried slides were stained with 4% (*v*/v) Giemsa solution (Sigma-Aldrich) in deionized water for 20 min. Untreated cells and 0.05 μg/mL mitomycin C (MMC, Sigma-Aldrich) were used as negative and positive controls. The samples were then scored as previously described [30, 31]. In short, 500 cells per slide were scored to evaluate cytostasis (replication index, RI) and the number of apoptotic, necrotic, and mitotic cells. The genotoxic potential of particle dispersions was evaluated by scoring the micronucleus frequency as number of 1000 binucleated cells containing one or more micronuclei (MN) per slide. The MN frequency in 1000 mononucleated cells was also considered in order to distinguish between aneuploidogenic and clastogenic effects [32]. Finally, nucleoplasmic bridges (NPB), a biomarker of DNA misrepair and/or telomere end-fusions, and nuclear buds (NBUD), a biomarker of elimination of amplified DNA and/or DNA repair complexes, were also scored in 1000 binucleated cells. Two slides per condition were examined in three independent experiments.

### Role of calcium and iron

To investigate the role of intracellular calcium and iron for the induction of MN, NPB, NBUD and cell death, cells were pretreated for 15 min with either 10 μM deferoxamine (Sigma-Aldrich), 5 μM verapamil (Sigma-Aldrich), 5 μM dantrolene (Sigma-Aldrich), or 0.1 μM BAPTA-AM (Molecular Probes, Life Technologies) and then co-exposed 48 h to 5 μg Ni/mL of Ni/NiO NPs or NiCl_2_. The concentrations of chelators/inhibitors were chosen based on previous cell viability experiments in order to avoid cytotoxic conditions.

### Chromosomal aberration assay

Structural aberrations at chromatid and/or chromosome level were assessed by chromosomal aberration assay (OECD 473**)**. BEAS-2B cells were seeded in 6-well plates. After 24 h, particles dispersions were directly added to the cell culture for 48 h at the final mass concentration of 5 μg Ni/mL. Untreated cells and 0.05 μg/mL MMC were used as negative and positive controls. In the last 3 h of cultures, 0.05 μg/mL demecolcine (Sigma-Aldrich) was added and cells were harvested, treated 5 min by 0.075 M KCl and washed three times with fresh cold fixative (methanol:acetic acid 3:1) and placed at − 20 °C overnight. Finally, cell suspensions were dropped onto clean and cold glass slides. The air-dried slides were stained with 4% (*v*/v) Giemsa solution (Sigma-Aldrich) in Sørensen’s buffer (pH 7.0) for 6 min. Chromosomal aberrations (CAs) were analyzed as described previously by Nymark et al. [33].

#### Statistical analysis

Statistical analysis was performed using GraphPad Prism 5 statistical software (GraphPad Inc.). One-way analysis of variance followed by Bonferroni’s multiple comparison test was used to test for significance between exposures and results are expressed as mean values ± standard error of mean (SEM) of three independent experiments (*n* = 3).

## Results

### Size, surface analysis and intrinsic ROS generation

**Fig. 1:**
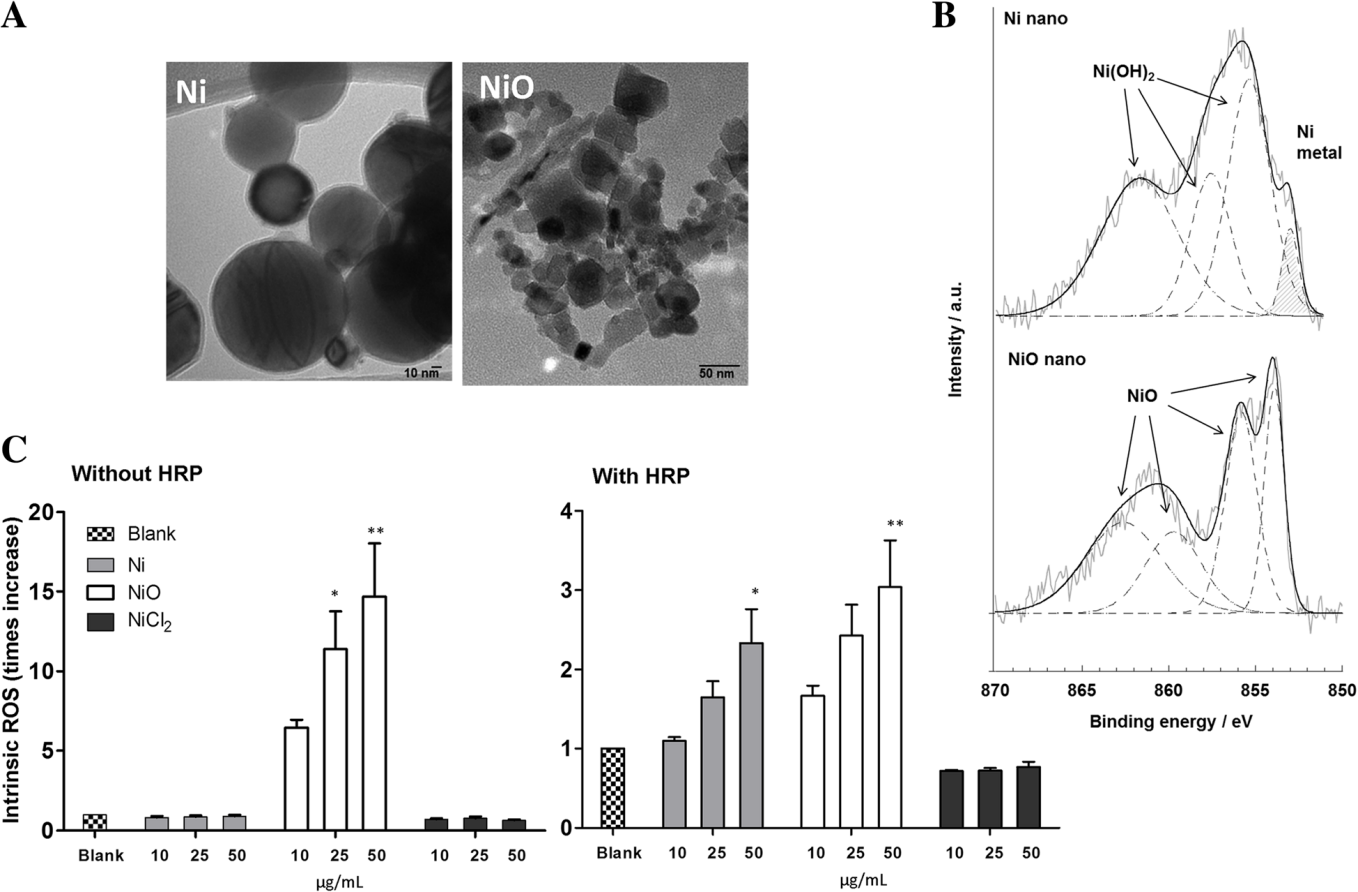
Nanoparticle characterization. **a** TEM images showing Ni NPs (left, bar indicates 10 nm) and NiO NPs (right, bar indicates 50 nm). **b** Fitted XPS spectra of Ni and NiO confirming the composition of the surface of the NPs. **c** Intrinsic ROS production measured using the DCFH assay without HRP (left) and with HRP (right)

In addition to the information from the manufacturer and our own characterization in previous studied [22, 24], we made new analyses to characterize the NPs. The TEM analysis showed Ni NPs to be of varying sizes but predominantly less than 100 nm, and NiO NPs less than 50 nm (Fig. 1a). Surface analysis using XPS showed for NiO NPs multiple split peaks of oxidized nickel at 853.7 ± 0.2 and 855.8 ± 0.2 eV (Ni 2p3/2) combined with characteristic satellite peaks and a sharp oxygen peak at 529.7 ± 0.2 eV (O 1 s) that correlate very well with literature findings for NiO [34, 35]. A metallic nickel signal (852.6 ± 0.1 eV) was evident on Ni NPs in addition to oxidized nickel (855.0 ± 0.2 eV) and satellite peaks that could be assigned to Ni (OH)2 (main O 1 s peak at 530.0 ± 0.2 eV) [34, 35]. The results clearly show the presence of a thin (< 5 nm) nickel (II)-rich surface oxide on metallic nickel. Fitted XPS spectra of the NPs are presented in Fig. 1b. To analyze the reactivity of the NPs, we tested the intrinsic ROS generating ability using the DCFH assay. The results showed NiO NPs to be highly reactive when the assay was performed without HRP whereas Ni and NiO showed rather similar (and much less) effects in the presence of HRP. No effects were observed for the NiCl_2_ (Fig. 1c).

### Rapid NP agglomeration and limited nickel release in serum-free cell culture medium

**Fig. 2:**
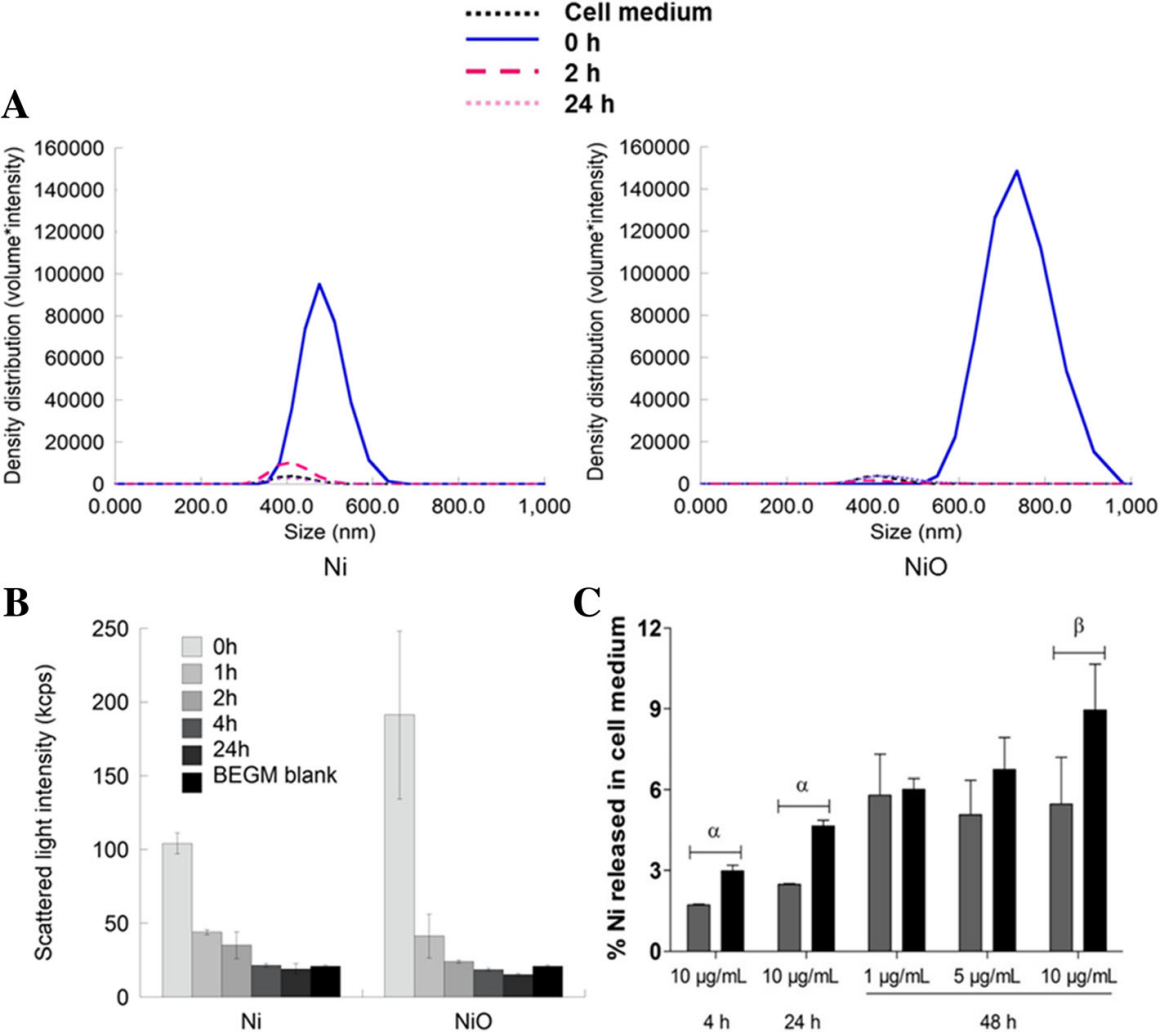
Nanoparticle characterization in cell medium. Nanoparticle behavior in cell medium (BEGM) was examined by photon cross-correlation spectroscopy (PCCS) and inductively coupled plasma mass spectrometry (ICP-MS). **a** The PCCS density distribution histograms are presented as density distribution by volume multiplied with corresponding scattered light intensities and indicate the hydrodynamic size distribution of the particles directly after dispersion (0 h), after 2 h, as well as 24 h at a concentration of 10 μg/mL, and (**b**) corresponding PCCS scattered light intensities. **c** The amount of Ni released in cell medium after 4, 24 and 48 h was determined by ICP-MS and expressed as % Ni released in cell medium from the total amount of added Ni (determined experimentally) for Ni (gray) and NiO (black)

In order to characterize the behavior of the NPs in cell medium (BEGM), we analyzed the hydrodynamic size and light scattering using photon cross-correlation spectroscopy (PCCS) as well as the dissolution/Ni release by means of ICP-MS. The hydrodynamic size measurements in solution (density distributions multiplied with corresponding scattered light intensities) showed that both Ni and NiO NPs agglomerate directly upon dispersion (0 h) in cell medium (BEGM), with an approximate average agglomerate size of 500 nm for the Ni NPs and 750 nm for NiO NPs (Fig. 2a). Negligible scattered light intensity was observed from the BEGM medium alone compared with intensities observed from solutions containing NPs. Signals and size distributions of the NP-containing samples were after 2 and 24 h of exposure similar to the BEGM medium, findings which indicate particle removal from solution by means of sedimentation. Significantly reduced scattered light intensities with time were observed for both the Ni and the NiO NPs (Fig. 2b). Following incubation with cell medium at 37 °C (10 μg Ni/mL) more Ni in solution (normalized to the total amount of added Ni) was determined for NiO NPs compared to Ni NPs at the tested time points 4 h, 24 h and 48 h (Fig. 2c). An increased amount of released Ni in solution (based on nickel content) was observed with time reaching levels of approx. 5% for the Ni NPs and 9% for the NiO NPs after 48 h. No significant differences were observed between the two NPs in terms of Ni release in solution after 48 h for the lower particle concentrations (1 and 5 μg Ni/mL).

### Ni and NiO NPs, but not ionic nickel species, are readily taken up by human lung cells

**Fig. 3:**
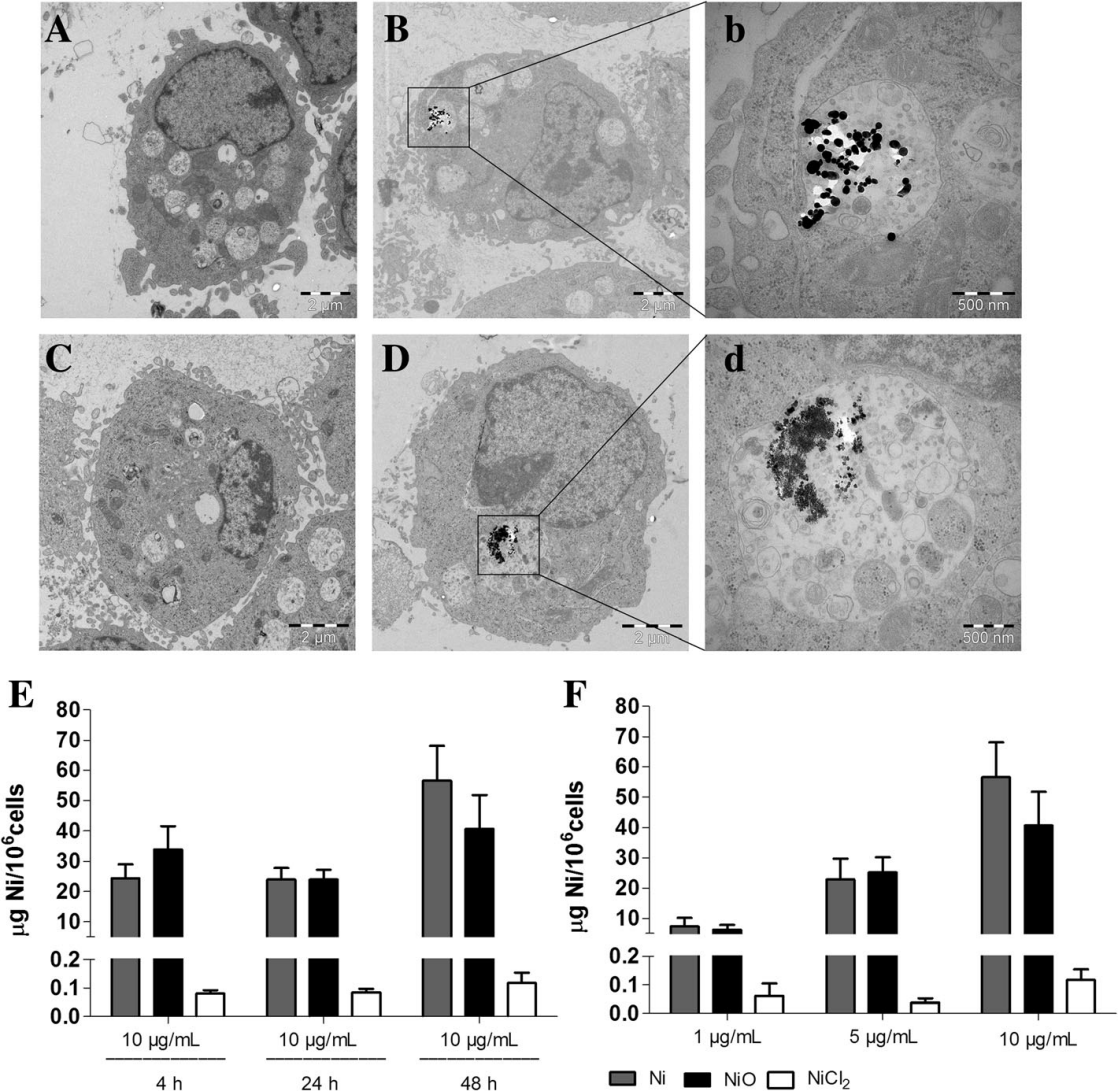
Intracellular localization and uptake. Cellular ultrastructural alteration and intracellular localization of Ni and NiO NPs in BEAS-2B cells was investigated by TEM (**a**, control cell; **b** with corresponding inserts **b**, Ni treated cell; **c** NiCl_2_ exposed cell; **d** with corresponding inserts **d**, NiO treated cell). After 48 h exposure to 10 μg Ni/mL of Ni (**b**) and NiO NPs (**d**), particles were taken up and contained within membrane-bound structures (**b** and **d**). The time-dependent cellular Ni content was investigated by ICP-MS after 10 μg Ni/mL (4 h, 24 h and 48 h) exposures (**e**) whereas the concentration-dependent uptake was studied following 1, 5 and 10 μg Ni/mL (48 h) exposures to Ni, NiO and NiCl_2_

To study cellular uptake and intracellular localization, TEM imaging as well as ICP-MS was conducted. TEM clearly showed that both Ni and NiO NPs were taken up by BEAS-2B cells and were contained within membrane-bound structures (Fig. 3a-d). Vacuoles reminiscent of autophagosomes and early autophagosomes evidenced by rough endoplasmic reticulum surrounding mitochondria were observed following exposure to both NPs as well as NiCl_2_. Exposure to Ni and NiO NPs rapidly increased the Ni cellular content as assessed by ICP-MS 4 h after exposure, reaching 40/60 μg of Ni per 10^6^ cells (thus higher total mass of NiO compared to Ni) following 48 h treatments whereas NiCl_2_ was not significantly taken up by BEAS-2B cells (Fig. 3e). The cell-associated Ni content increased with increasing mass concentrations of Ni and NiO NPs (Fig. 3f).

### NiO NPs induce more apoptosis in BEAS-2B cells compared to Ni NPs and ionic Ni

**Fig. 4:**
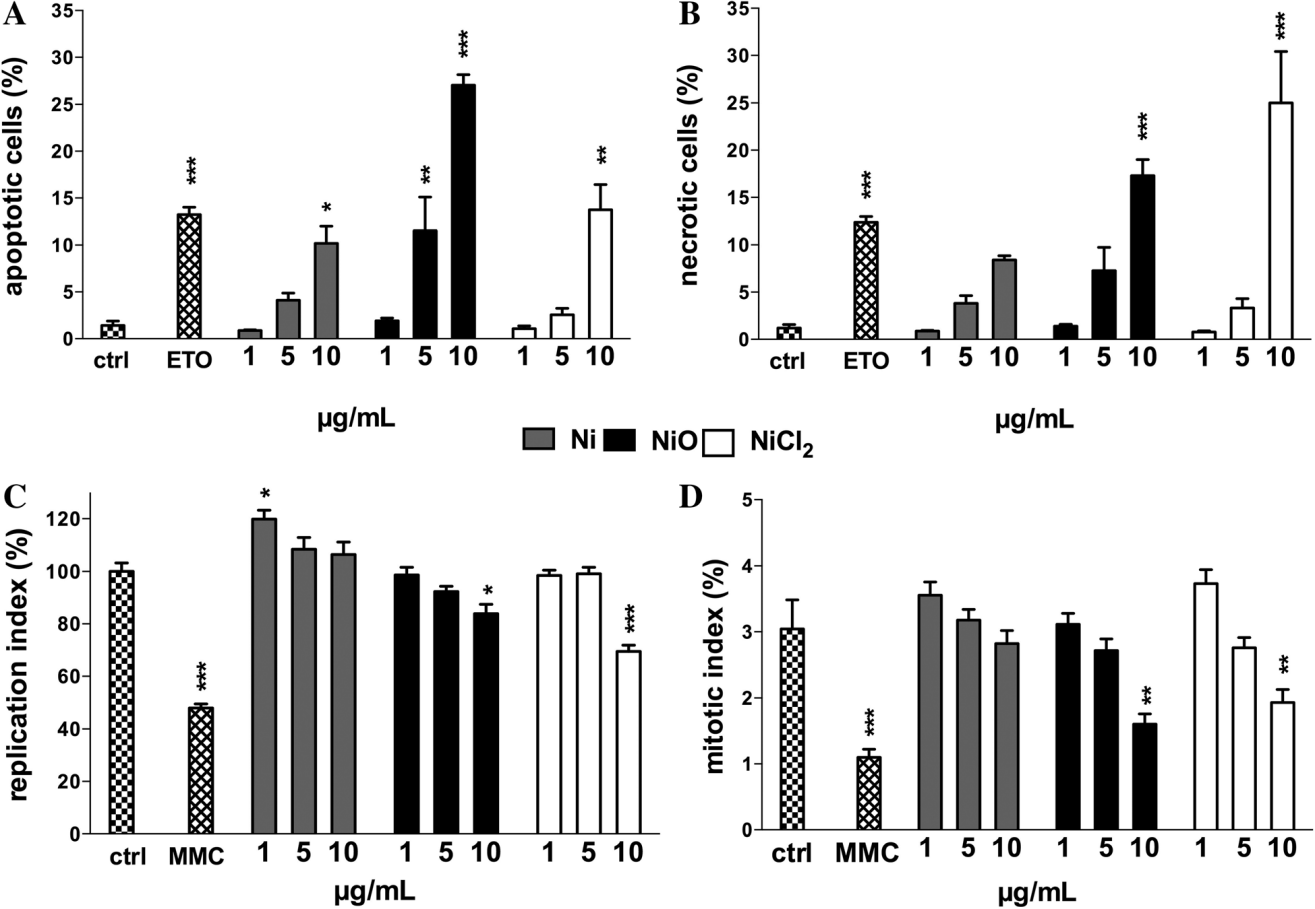
Cytotoxicity and cytostasis. The percentage of apoptotic (**a**) and necrotic cells (**b**) as evaluated by annexin V/PI staining (ETO, Etoposide 0.2 μg/mL was used as positive control). The percentage of replication (**c**) and mitotic (**d**) indices as evaluated by CBMN Cyt assay (MMC, mitomycin C 0.05 μg/mL was used as positive control). Results are presented as mean ± SEM from three independent experiments (*n* = 3). *, *p* < 0.05; **, *p* < 0.01; ***, *p* < 0.001

To obtain a comprehensive view of the cytotoxic/cytostatic effects of the different exposures, we performed annexin V/PI staining (necrosis/apoptosis) and scoring of cells microscopically (as performed in the CBMN Cyt assay). Following Ni and NiO NP exposures, the percentage of apoptotic cells increased in a dose-dependent manner and NiO was clearly most effective leading to approx. 25% apoptotic cells in the highest concentration (10 μg Ni/mL) (Fig. 4a). The fraction of necrotic cells also increased particularly in the highest concentration (10 μg Ni/mL) of NiO NPs and NiCl_2_ (Fig. 4b). The same trend regarding apoptosis and necrosis was also observed from cells scored microscopically in the CBMN Cyt assay (Additional file 1: Figure S1). The microscopic analysis also showed, interestingly, that the lowest concentration of Ni NPs increased the replication index when compared to the control (Fig. 4c). In contrast, 10 μg Ni/mL of NiO NPs and NiCl_2_ showed a significant cytostatic effect (Fig. 4c) and also reduced the mitotic index (Fig. 4d). Taken together, the data showed NiO NPs to be more cytotoxic compared to Ni NPs. Furthermore, a low dose of Ni NPs was found to increase cell replication.

### NiO NPs cause more DNA strand breaks, intracellular ROS and Ca^2+^ compared to Ni NPs and ionic Ni

**Fig. 5:**
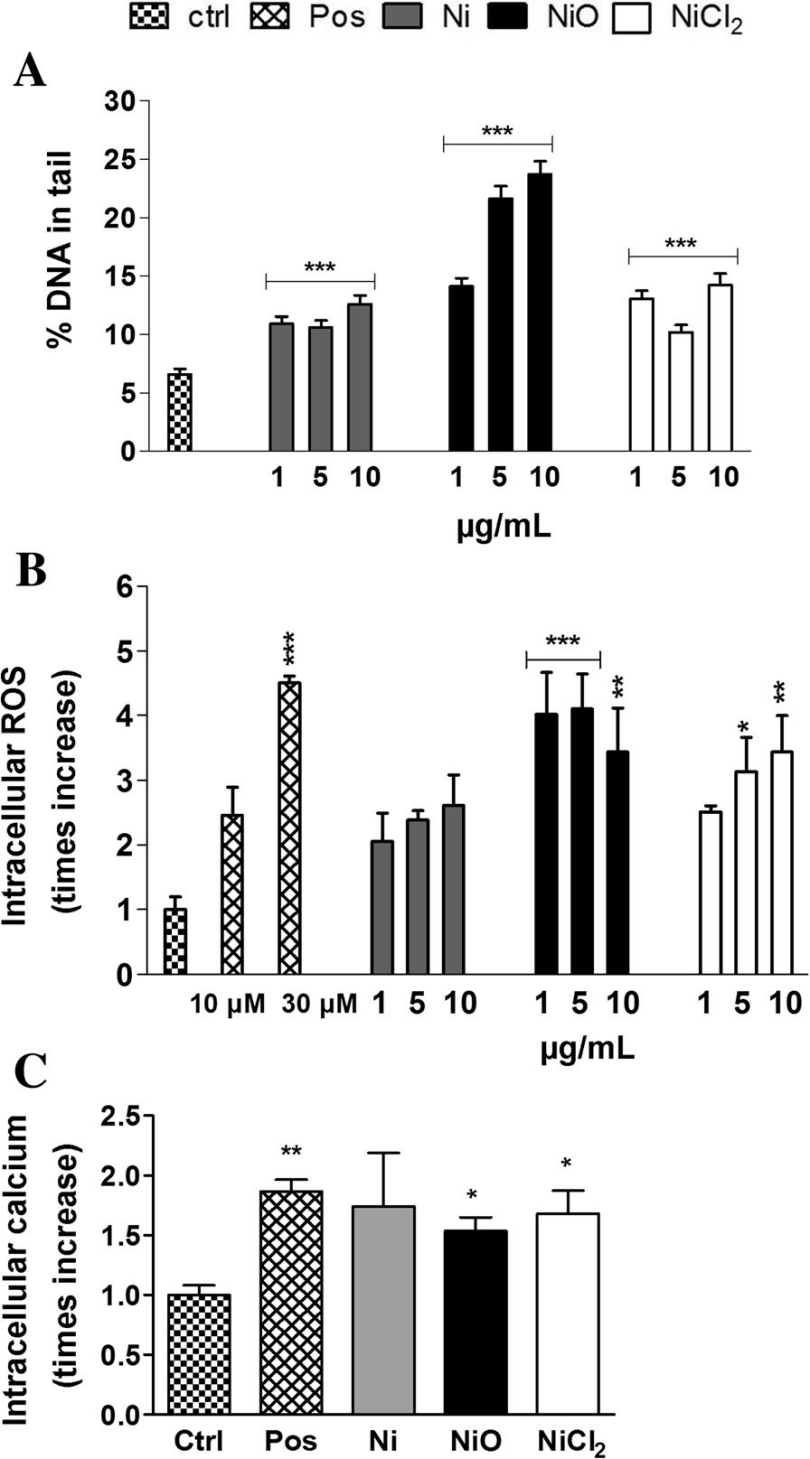
DNA strand breaks, intracellular ROS and calcium. **a** DNA damage expressed as % DNA in tail following 48 h Ni, NiO and NiCl_2_ (μg Ni/mL) exposure of BEAS-2B cells, (**b**) ROS increase calculated as mean slope per min from the first 2 h exposures and normalized to the unexposed control and (**c**) intracellular calcium measured using the probe Fluo-4 AM (Fluo-4-acetoxymethyl ester, 5 μM) following 2 h exposure. Results are presented as mean ± SEM from three independent experiments (n = 3). H_2_O_2_ (25 μM) was used as positive control in comet assay, tert-butyl hydroperoxide (TBP, 10 and 30 μM) was used as positive control in ROS level studies and calcium ionophore A23187 (2.5 μM) as positive control for intracellular calcium (*p* < 0.001). *, *p* < 0.05; **, *p* < 0.01; ***, *p* < 0.001

Next, we investigated the ability to cause DNA strand breaks (comet assay) and intracellular ROS formation (DCFH-DA assay). All exposures increased the DNA damage compared to control and NiO was the most potent causing >3 times increase in DNA damage in the two highest doses (Fig. 4b). Ni and NiCl_2_ caused modest effects without clear dose-response. NiO NPs were also the most potent inducer of intracellular ROS with a significant increase in all doses tested (Fig. 5a). Increased levels were also noted for NiCl_2_ but not for Ni NPs. Similarly, NiO NPs and NiCl_2_ caused a statistically significant increase in intracellular Ca^2+^, whereas Ni NPs showed more variation among the experiments leading to a non-significant increase (Fig. 5c).

### All Ni exposures cause chromosomal damage and rearrangements

**Fig. 6:**
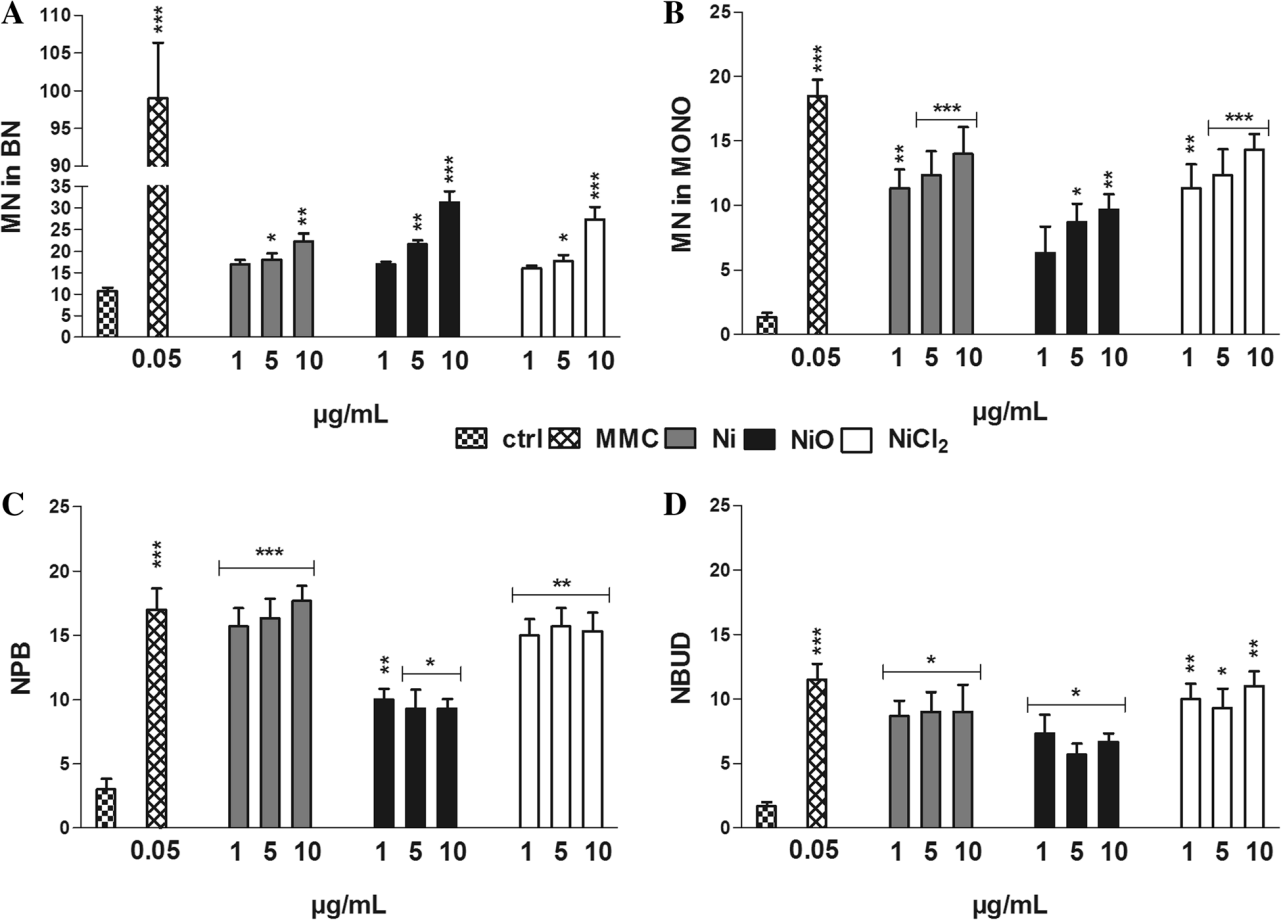
Chromosomal damage and rearrangements. Micronuclei in binucleated cells (**a**), micronuclei in mononucleated cells (**b**), nucleoplasmic bridges (**c**) and nuclear buds (**d**) frequencies as evaluated by CBMN Cyt assay following 48 h exposure of BEAS-2B cells to Ni, NiO or NiCl_2_ (μg Ni/mL). Results are presented as mean ± SEM from three independent experiments (n = 3) of the different kind of damage in 1000 binucleated or mononucleated cells. Mitomycin C (MMC, 0.05 μg/mL) was used as positive control. *, *p* < 0.05; **, *p* < 0.01; ***, *p* < 0.001

In order to acquire more in-depth insight into genotoxicity, we analyzed the frequency of micronuclei (MN), nucleoplasmic bridges (NPB), a biomarker of DNA misrepair and/or telomere end-fusions, nuclear buds (NBUD), a biomarker of elimination of amplified DNA and/or DNA repair complexes as well as chromosomal aberrations according to OECD accepted protocols (OECD 487, OECD 473). The frequency of MN in binucleated cells was significantly higher compared to control cells for the two highest concentrations tested for all three Ni exposures. NiO NPs were most potent causing approx. 3% MN in binucleated cells compared to 1% in the control (Fig. 6a). Ni NPs and NiCl_2_ were more effective than NiO NPs in inducing micronuclei in mononucleated cells, indicating aneuploidogenic activity (Fig. 6b). Similarly, Ni NPs and NiCl_2_ increased NPB and NBUD frequencies to a higher extent as compared with NiO NPs (Fig. 6c and d). Analysis of chromosomal aberrations showed that Ni NPs and NiCl_2_ significantly increased the rate of chromatid-type aberrations and induced both inter- and intra-arm exchanges (See Additional file 1: Table S1). All treatments induced chromosome-type aberrations and NiO NPs were the most effective in inducing chromosome breaks and acentric fragments whereas Ni NPs and NiCl_2_ were the most potent in inducing the formation of dicentric chromosomes as well as endo-reduplications (duplication of nuclear genome in the absence of cell division) (See Additional file 1: Table S1 and Figure S2). Untreated BEAS-2B cells showed a modal chromosome number (45.9 ± 0.6) whereas all exposures induced various degree of ploidy such as trisomy, and to a lesser extent monosomy, involving particularly chromosomes 1, 3, 14, 20 and 21. The mitotic index was slightly increased in Ni NP exposed cells and decreased following both NiO NPs and to a lower extent NiCl_2_ exposures, albeit not significantly compared to the untreated control. Taken together, all three Ni exposures significantly induced chromosomal aberrations in the BEAS-2B lung cells.

### Ni-induced genome instability and cytotoxicity are calcium- and iron dependent

**Fig. 7:**
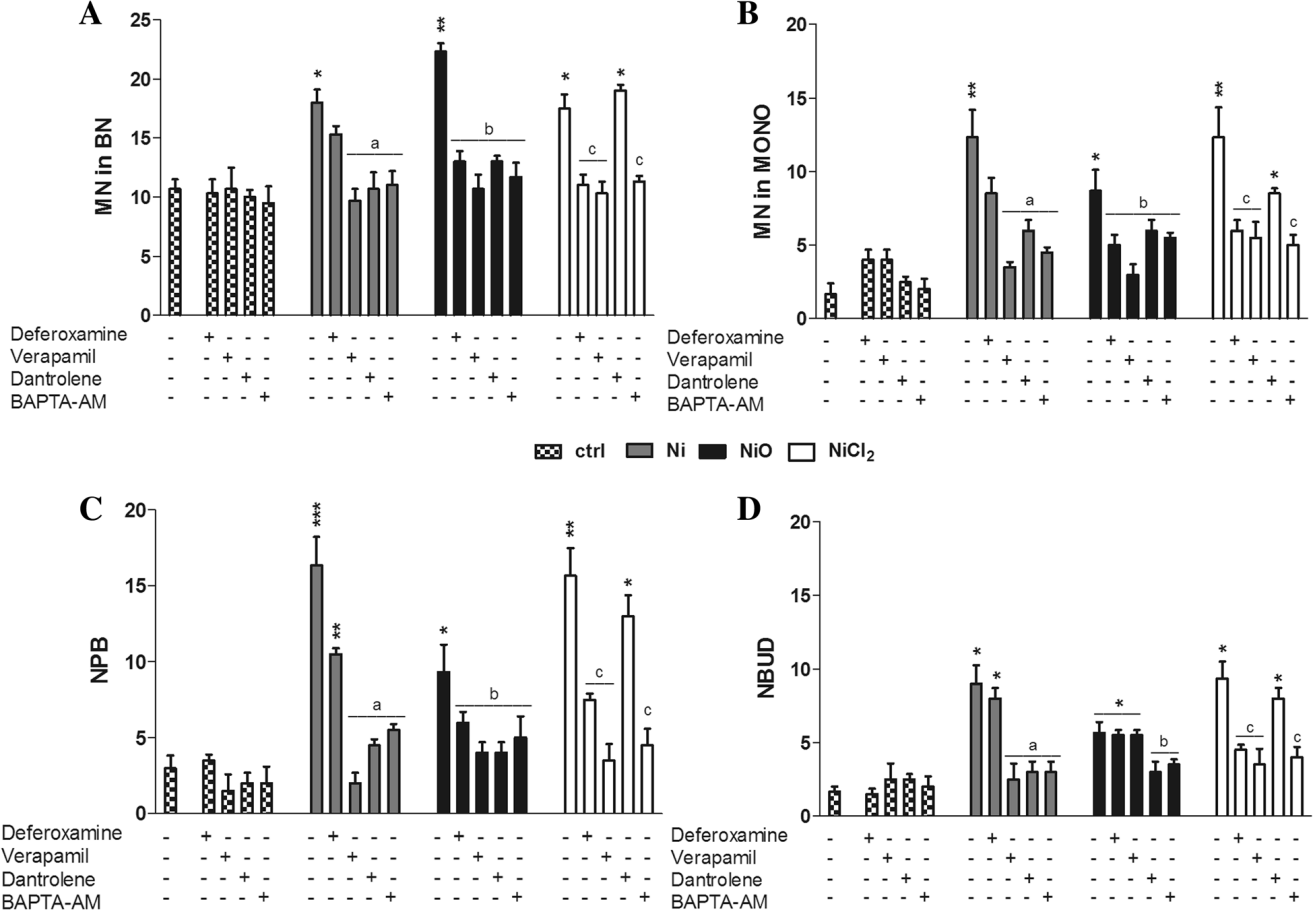
Effect of iron chelator and calcium modulators on chromosomal damage and rearrangements. Micronuclei in binucleated cells (**a**), micronuclei in mononucleated cells (**b**), nucleoplasmic bridges (**c**) and nuclear buds (**d**) frequencies as evaluated by CBMN Cyt assay following co-exposures of 5 μg Ni/mL Ni, NiO or NiCl_2_ with deferoxamine, verapamil, dantrolene or BAPTA-AM for 48 h. Results are presented as mean ± SEM from three independent experiments (n = 3) of the different kind of damage in 1000 binucleated or mononucleated cells. *, *p* < 0.05; **, *p* < 0.01; ***, *p* < 0.001. Asterisks represent the statistical significance compared to the control whereas a, b and c represent the significance of the chelator/modulator compared to Ni-, NiO-, NiCl_2_- treated cells, respectively

**Fig. 8:**
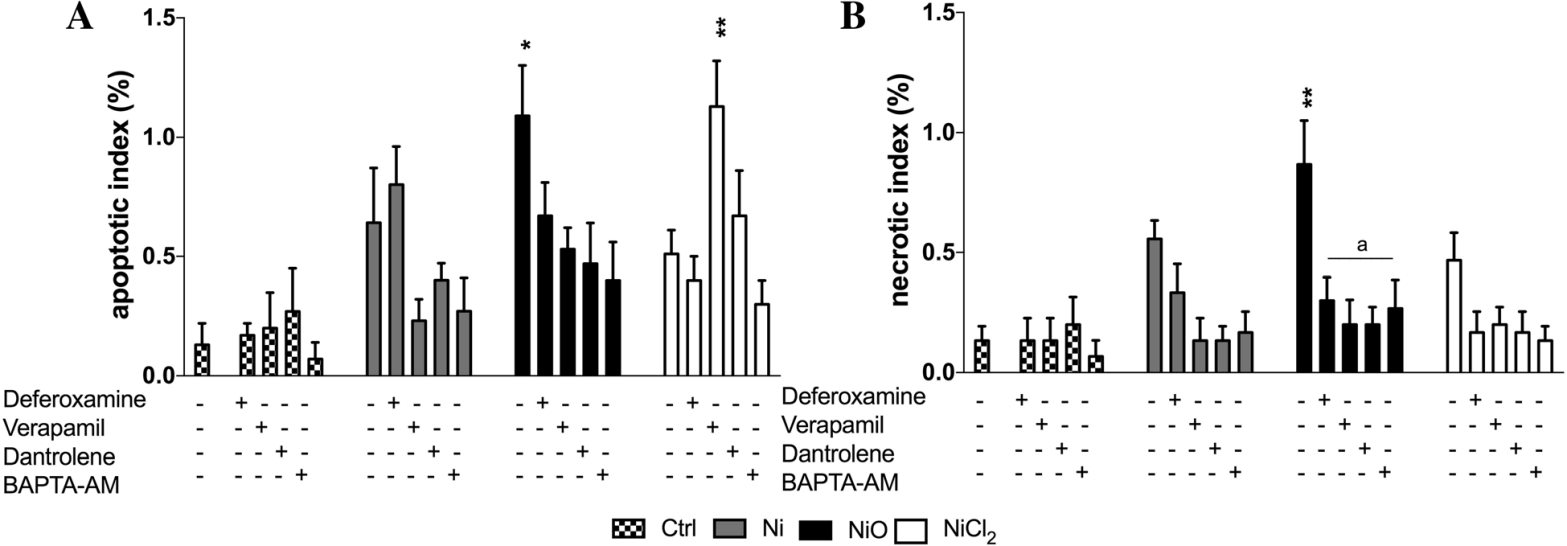
Effect of iron chelator and calcium modulators on cytotoxicity. The percentage of apoptotic (**a**) and necrotic (**b**) cells as evaluated by CBMN Cyt assay following co-exposures of 5 μg Ni/mL Ni, NiO or NiCl_2_ with deferoxamine, verapamil, dantrolene or BAPTA-AM for 48 h. Results are presented as mean ± SEM from three independent experiments (n = 3). *, *p* < 0.05; **, *p* < 0.01; ***, *p* < 0.001

Finally, we attempted to identify possible underlying mechanisms for the chromosomal damage observed. Given the results showing clear chromosome damage from all three Ni exposures despite the differences in uptake (no measurable uptake of NiCl_2_), we decided to evaluate mechanisms that are independent on uptake such as effects on calcium signaling and iron metabolism that may be mediated via the plasma membrane or are related to oxidative stress [36]. Co-exposure of BEAS-2B with the iron chelator deferoxamine followed by treatment with 5 μg Ni/mL of Ni/NiO NPs or NiCl_2_ significantly reduced the NiO- and NiCl_2_-induced increase in MN to levels near the control value (Fig. 7a and b) whereas exposure to Ni NPs was less affected. Similar results were also observed for formation of NPB and NBUD (Fig. 7c and d). For the calcium modulators, co-exposure of BEAS-2B with verapamil, an inhibitor of calcium uptake through the plasma membrane, or BAPTA-AM, a cell-permeable Ca^2+^ chelator, followed by treatment with 5 μg Ni/mL of Ni/NiO NPs or NiCl_2_ significantly reverted genotoxicity to control values for all Ni exposures. Co-treatment with dantrolene, an antagonist of ryanodine (Ry) receptors preventing Ca^2+^ release from the endoplasmic reticulum (ER), protected against NPs-induced genotoxicity but not the NiCl_2_-induced genotoxicity (Fig. 7). In addition, similar protective effects were observed for NiO-induced apoptosis and necrosis (Fig. 8a and b).

## Discussion

Despite considerable progress in understanding the toxicological effects of NPs, less is known about their impact at the chromosomal level and underlying mechanisms are poorly understood. In this study, we investigated the ability of Ni and NiO NPs to induce DNA and chromosomal damage in BEAS-2B cells and we compared these effects with those of ionic Ni from soluble NiCl_2_. To discern the underlying mechanism, we studied cell uptake and explored the role of calcium and iron by using a range of inhibitors and chelators. The results revealed chromosomal damage by Ni and NiO NPs as well as Ni ionic species and calcium signaling was implicated in this process.

**Fig. 9:**
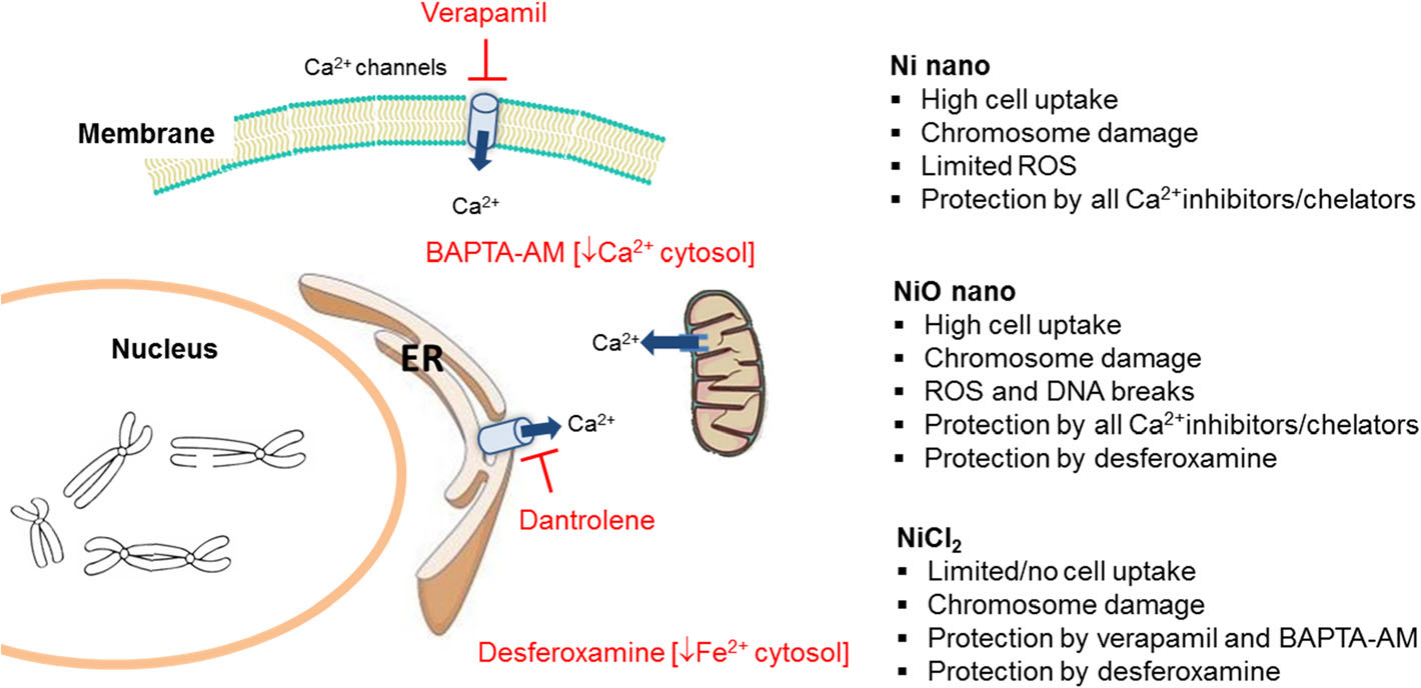
Summary of main results. Ni NPs, NiO NPs and ionic Ni (NiCl_2_) caused chromosomal damage that was dependent on Ca^2+^. Changes in cytosolic Ca^2+^ can take place by entry through plasma membrane Ca^2+^ channels, release of Ca^2+^ from the endoplasmic reticulum (ER) or release from the mitochondria via opening of the mitochondrial permeability transition pore

We initially characterized the reactivity of the NPs and found a high intrinsic ROS generating ability of NiO as also has been shown previously when compared to other metal oxide NPs [37, 38]. ROS was analyzed using DCFH that can be oxidized to fluorescent DCF by hydroxyl radicals and peroxynitrite, as well as by H_2_O_2_ in the presence of peroxidases such as HRP. The high fluorescence observed without HRP for NiO NPs suggests formation of hydroxyl radicals and peroxinitrite, in contrast to Ni NPs only showing activity in the presence of HRP thus suggesting formation of H_2_O_2,_ The ROS production was particle-specific since no effects were observed for ionic Ni. We noted that the NPs agglomerated in cell medium and Ni was released from both Ni and NiO NPs in a time-dependent manner corresponding to approx. 2–9% of the total mass Ni after 4–48 h, in line with previous investigations [22, 24]. The somewhat higher release from NiO than Ni is expected and related to the barrier properties of a thin passive surface oxide of the Ni NPs (shell-core structure) of different characteristics compared with the NiO NPs lacking such a surface barrier and metallic core [21, 22]. The higher surface area (BET) for the NiO NPs could also be an explanation since this reflects differences in particle size and/or different porosity at dry conditions. The difference in available surface area will likely be less pronounced in solution since both NPs rapidly agglomerated. Next, we considered the cellular dose of the exposures. The importance of cellular dosimetry of NPs for in vitro toxicity studies is clearly well established [39] and recently, a new model that can be used to estimate delivery of both particles and released ionic species to cells has been developed [40]. However, instead of modeling the dose, we measured the dose, i.e., the NPs taken up or firmly attached to the cells, by using ICP-MS. High uptake was observed for the NPs whereas no clear increase in Ni was observed in cells exposed to NiCl_2_. The uptake for ionic Ni is expected to be low, but it has been reported that Ni ionic species may enter cells via cell membrane transporters, such as the DMT1, or via calcium channels [9]. Despite the apparent lack of uptake of Ni ions/complexes, an increase in oxidative stress, DNA strand break formation and chromosomal damage was not only observed for the NPs, but also for NiCl_2_. These findings led us to search for mechanisms that, at least partly, may be explained by interactions with the cell membrane/receptors. Previous studies have shown that Ni^2+^ ions block calcium channels and Ni ionic species/complexes have also been found to lead to increased intracellular Ca^2+^, possibly via a compensatory intracellular release of Ca^2+^ from intracellular stores [41]. Furthermore, Ni has been shown to elicit Ca^2+^-dependent interleukin (IL)-8 gene activation in the human monocytic cell line THP-1, as evidenced by the inhibitory action of the Ca^2+^ channel inhibitor, nifedipine [42]. The authors speculated on the mechanisms by which divalent cations such as Ni^2+^ enter cells and suggested that calcium channels may be transducers of Ni due to metal ion ‘mimicry’. The involvement of Ca^2+^ signaling in the present model appeared plausible since we noted an increase in intracellular Ca^2+^ as a result of the Ni exposures. To test the involvement of Ca^2+^ signaling, we employed various modulators of calcium signaling: verapamil, an inhibitor of voltage-dependent Ca^2+^ channels in the plasma membrane, the intracellular Ca^2+^ chelator, BAPTA-AM, as well as dantrolene, an antagonist of ryanodine (Ry) receptors in the ER (Fig. 9). We found that all three Ca^2+^ modulators clearly prevented the chromosomal damage induced by the NPs, thus providing evidence for a role of Ca^2+^ in the genotoxicity of Ni/NiO NPs. However, in the case of NiCl_2_, dantrolene was not protective, suggesting that release of Ca^2+^ from intracellular stores was important for the genotoxicity induced by the NPs, but not by NiCl_2_. Hence, our studies have revealed a particle-specific mechanism, though the final endpoint (chromosomal damage) is not necessarily unique for particles. Other studies have also shown an increase in intracellular Ca^2+^ following exposure to high doses of soluble green nickel carbonate hydroxide (Ni_5_(CO_3_)_2_(OH)_6_·4H_2_O) [43] or to other NPs such as CdSe quantum dots [44]. In addition to the effect on Ca^2+^ modulation, treatment with the iron chelator, deferoxamine was also protective for NiO NPs and NiCl_2_, i.e., the same exposures causing increased intracellular ROS. The protective effect of deferoxamine suggests a mechanism by which Ni triggers increased levels of intracellular iron that may take part in Fenton-like reactions causing oxidative stress and DNA damage. Deferoxamine has previously been suggested to prevent DNA-protein crosslink formation induced by poorly soluble Ni_3_S_2_ [45]. Furthermore, in studies on lymphocytes exposed to Ni_5_(CO_3_)_2_(OH)_6_·4H_2_O, M’Bemba-Meka and co-workers showed a protective effect of both deferoxamine and calcium modulators for the formation of DNA strand breaks and sister-chromatid exchange [46, 47]. In our study, we also found protection from NiO-induced apoptosis and necrosis from all inhibitors/chelators in line with the fact that cellular Ca^2+^ overload, or perturbation of intracellular Ca^2+^ compartmentalization, can cause cytotoxicity and trigger either apoptotic or necrotic cell death [48]. Taken together, the current data along with other recent studies suggest the involvement of calcium and iron in the toxicity of soluble Ni compounds as well as of Ni and NiO (nano) particles.

In the nano (geno) toxicological field, a critical question concerns the importance of direct DNA interactions versus non DNA-reactive mechanisms. Using mechanism-specific reporter cell lines, we recently showed that oxidative stress appeared to be a main toxic mechanism of Ni and NiO NPs/ionic Ni species, and none of these exposures induced the reporter related to direct DNA damage and stalled replication forks, indicating that they are not direct DNA reactive [24]. In the current study using BEAS-2B cells, Ni NPs showed some divergent effects compared to NiO NPs, such as an increased proliferation, in line with previous findings [22], effects similar to aneugen-like spindle poisons, and endoreduplications (duplication of nuclear genome in the absence of cell division). Interestingly, endoreduplications and multinuclear cells as well as disruption of tubulin filaments were recently reported in A549 lung carcinoma cells following exposure to magnetite NPs [49]. In general, interference with the cytoskeleton organization/tubulin filaments appears plausible in cells with high content of NPs [50–52]. However, the lack of uptake of NiCl_2_, as evidenced in the present study, indicates that cell surface proteins/receptors may also be involved, as proposed for Cd^2+^ (another Ca^2+^ ‘mimetic’) [53]. Another receptor that could potentially be involved is TRPV4, a Ca^2+^ permeable non-selective cation channel that acts as a regulator of both microtubules and actin [54]. Interestingly, silica NPs were recently shown to inhibit TRPV4 activation in cultured human airway epithelial cells 16HBE [55]. Taken together, the involvement of plasma membrane receptors versus intracellular effects in relation to the effects of Ni and NiO NPs, as well as Ni ionic species, need to be explored further. It is also important to note that the toxicological effects for NPs are expected to be more severe than for soluble Ni species, due to higher lung retention. For instance, Shinohara et al. showed recently that after intratracheal administration in rats, 40–60% of the dose remained in the lungs after 90 days for poorly soluble spherical NiO NPs whereas <0.3% remained in case of the soluble NiO nanowires [56].

## Conclusion

In conclusion, the present study has shown that Ni and NiO NPs as well as Ni ionic species triggered chromosomal damage in a human lung cell line and has provided evidence for a mechanism that does not necessarily require cellular uptake, but depends on the modulation of intracellular calcium and iron. NiO-induced cell death in the present model was also shown to be calcium-dependent.

## Additional file

Additional file 1: **Table S1.** Chromatid- and chromosome-type aberrations and mitotic index. **Figure S1.** Apoptotic and Necrotic indices by CBMN Cyt assay. **Figure S2.** Representative metaphases of BEAS-2B cells (DOCX 267 kb).
