# Supplementary material for: Calcium-dependent cyto- and genotoxicity of nickel metal and nickel oxide nanoparticles in human lung cells
Source: Part Fibre Toxicol. 2018 Jul 17;15:32. doi: 10.1186/s12989-018-0268-y (PMC6050732; doi:10.1186/s12989-018-0268-y)
Supplement: Supplementary file 1 — Table S1. Chromatid- and chromosome-type aberrations and mitotic index. Figure S1. Apoptotic and Necrotic indices by CBMN Cyt assay. Figure S2. Representative metaphases of BEAS-2B cells (DOCX 267 kb). [file 12989_2018_268_MOESM1_ESM.docx]

**Additional files**

**Table S1. Chromatid- and chromosome-type aberrations and mitotic index.** Mean values (SD) of the percentage of metaphases with chromatid-, chromosome-type aberrations and mitotic index following exposure of BEAS-2B cells to 5 µg Ni/mL of Ni, NiO NPs and NiCl_2_. * *p*<0.05 (Kruskal–Wallis test). The positive control (mitomycin C, 0.05 µg mL^-1^) induced a significant increase in chromatid-type aberrations (*p*<0.01), chromosome-type breaks and acentric fragments (p<0.05), except for dicentric chromosomes and endo-reduplications.

|  | Chromatid-type | | | | | Chromosome-type | | | | | | MI |
| --- | --- | --- | --- | --- | --- | --- | --- | --- | --- | --- | --- | --- |
|  | Gaps | Breaks | Ex-changes | **Total w/o gaps** | Breaks | | Dicentric | Endo-reduplic. | Acentric fragments | **Total** |  | |
| Ctrl | 2.0  (0.1) | 3.1  (0.2) | 0 | **3.1**  **(0.2)** | 2.0  (0.1) | | 2.0  (0.4) | 0 | 0 | **4.0**  **(0.3)** | 4.0  (1.0) | |
| Ni | 9.8  (0.6) | 6.5  (0.4) | 3.2  (0.4) | **9.7***  **(0.4)** | 4.3  (0.3) | | 6.4  (0.6) | 5.1  (1.2) | 6.8  (1.2) | **22.6***  **(1.0)** | 5.9  (0.8) | |
| NiO | 10.4  (0.8) | 4.2  (0.2) | 0 | **4.2**  **(0.1)** | 9.7  (0.8) | | 4.2  (0.2) | 0 | 12.9  (2.2) | **26.8***  **(1.2)** | 3.0  (0.4) | |
| NiCl_2_ | 8.2  (0.6) | 5.4  (0.3) | 3.8  (0.8) | **9.2***  **(0.6)** | 3.8  (0.6) | | 5.9  (0.6) | 3.9  (0.7) | 5.9  (0.6) | **19.5***  **(0.8)** | 3.6  (0.6) | |

**Supplementary figures**

**Figure S1. Apoptotic and Necrotic indices by CBMN Cyt assay**

**
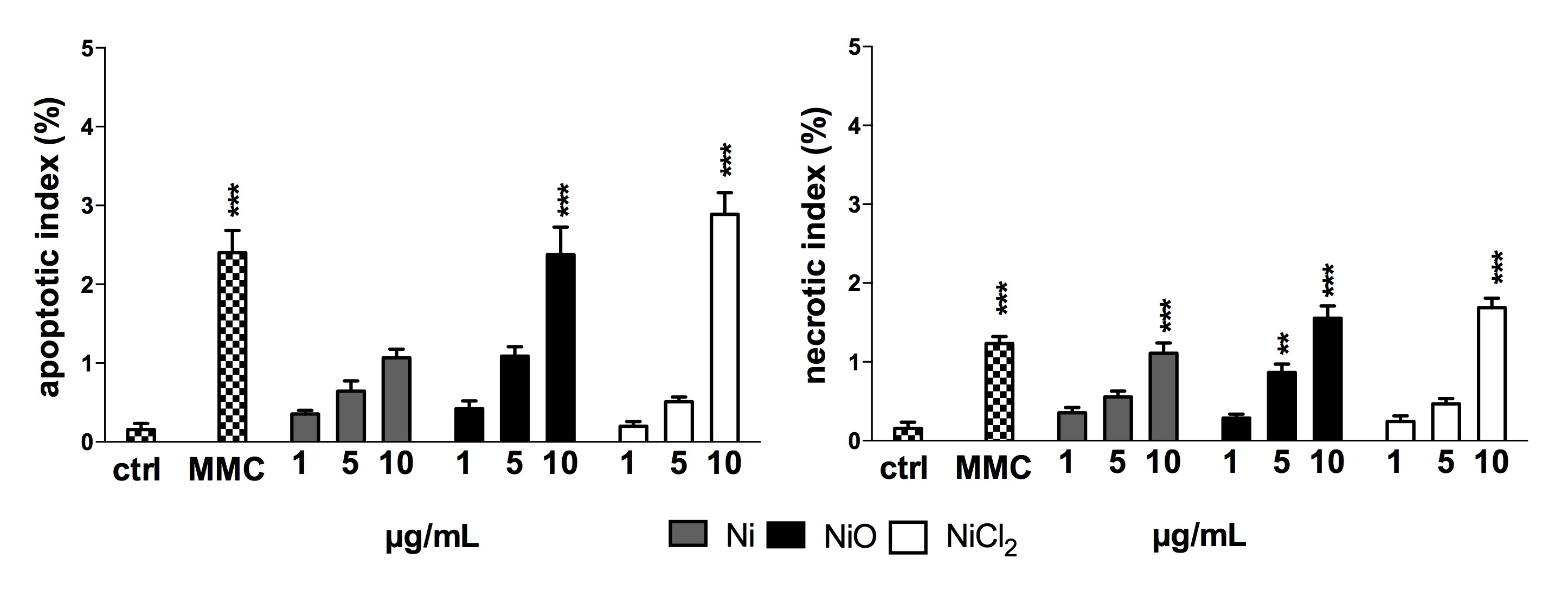
**

**Figure S1.** The percentage of apoptotic and necrotic cells as evaluated by CBMN Cyt assay following 48 h Ni, NiO or NiCl_2_ exposure of BEAS-2B cells (MMC, Mitomycin C 0.05 µg/mL was used as positive control). Results are presented as mean±SEM (n=3). **, *p*<0.01; ***, *p*<0.001.

**Figure S2. Metaphases of BEAS-2B**


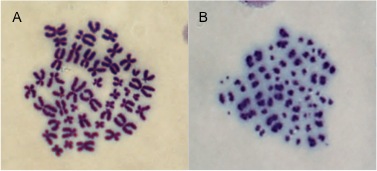


**Figure S2. Representative metaphases of BEAS-2B cells.** Metaphase of untreated cells (A) and endoreduplicated chromosomes (B).
